# Supplementary material for: High-throughput mechanophenotyping of multicellular spheroids using a microfluidic micropipette aspiration chip
Source: Lab Chip. 2023 Feb 16;23(7):1768–78. doi: 10.1039/d2lc01060g (PMC10045894; doi:10.1039/d2lc01060g)
Supplement: LC-023-D2LC01060G-s001 [file LC-023-D2LC01060G-s001.pdf]

Electronic Supplementary Material (ESI) for Lab on a Chip.
This journal is © The Royal Society of Chemistry 2023

High-throughput mechanophenotyping of multicellular spheroids using a microfluidic micropipette aspiration chip – Supplementary Material

Ruben C. Boot,[a] Alessio Roscani,[a] Lennard van Buren,[b] Samadarshi Maity,[a] Gijsje H. Koenderink, [b] and Pouyan E. Boukany[a]

[a] Department of Chemical Engineering, Delft University of Technology, Delft, The Netherlands.

[b] Department of Bionanoscience, Kavli Institute of Nanoscience Delft, Delft University of Technology, Delft, The Netherlands

Electronic Supplementary Information (ESI)

Fitting viscoelastic response to different spring-and-dashpot models

We fitted four different spring-and-dashpot models to the viscoelastic response of spheroids aspirated on our microfluidic chip, and compared them by their coefficient of determination (R^2). We tested the *Kelvin-Voigt model* (K-V, spring and dashpot in parallel), y = A × (1 − exp(B × t)), the *Standard Linear Solid model* (SLS, a spring followed by a spring and dashpot in parallel), y = A − B × (1 − exp(C × t)), the *Standard Linear Liquid model* (SLL, a dashpot followed by a spring and dashpot in parallel), y = A × (1 − exp(B × t)) + C × t, and the *modified Maxwell model* (MM, a dashpot in series with a Kelvin-Voigt element, consisting out of a spring in parallel with a spring and dashpot in series), y = A × (1 − B × exp(C × t)) + D × t. We calculated the coefficient of determination (R^2) value for each model and spheroid type using Python (see Table S1).

Glass capillary micropipette aspiration

HEK293T spheroids were aspirated using pipettes with a diameter of 65±5 µm, fabricated by pulling borosilicate glass pipettes (Harvard Apparatus, 1 mm OD, 0.5 mm ID) with a laser-based puller (Sutter Instruments Co Mode P-2000) and cutting them with a quartz tile. Cell adhesion to the pipette walls was prevented by incubating the pipettes in 2 mg/mL PolyEthyleneGlycol-PolyLysine (PLL(20)-g[3.5]-PEG(2)/PEG(3.4)-Biotin(20%), SuSos AG, Dubendorf, Switzerland) in MRB80 solution (80 mM piperazine-N,N'-bis(2-ethanesulfonic acid) (Pipes), pH 6.8, 4 mM $MgCl_2$, 1 mM EGTA [Sigma]) for 30 minutes. Spheroids were suspended in a CO_2-equilibrated medium and kept in a custom-made sample chamber. The chamber was constructed by adhering two microscope slides to a custom-made aluminum spacer of 3 mm thickness using vacuum grease (Beckman Coulter). The pipette was introduced into the chamber, aligned with a spheroid and an aspiration pressure was attained by vertically displacing a water reservoir connected to the pipette using a vertical translational stage (LTS300, Thorlabs). Spheroids were aspirated using a pressure ΔP = 5 cmH_2O and visualized on an inverted microscope (Nikon Eclipse TI) with a 10x air objective. After aspiration, the pressure gradient was removed and the retraction of the tongue was recorded. The creep advancement of the tongue was recorded with an ORCA Flash 4.0 digital camera using a 1s interval for a total of 10 minutes, of which 5 minutes corresponded to aspiration and the next 5 minutes to retraction. Data was obtained for two independent experiments, which were performed at room temperature. As the experimental set-up did not include a heating stage to keep the experimental chambers at the physiological temperature of 37 °C, aspiration of spheroids was only performed in the first hour after they came out of the incubator.

The critical pressure ΔP_c to aspirate the spheroids was derived from:

$$\Delta P_c = \Delta P \frac{\dot{L}_R}{\dot{L}_R + \dot{L}_A}, \qquad \text{(S1)}$$

where $\dot{L}_R$ and $\dot{L}_A$ are the retraction and aspiration flow rates respectively[1,2].

References

1 K. Guevorkian, M.-J. Colbert, M. Durth, S. Dufour and F. Brochard-Wyart, Physical Review Letters, 2010, 104, 1–4.

2 M. S. Yousafzai, V. Yadav, S. Amiri, Y. Errami, S. Amiri and M. Murrell, Physical Review Letters, 2022, 128, 48103.

Supplementary Figures

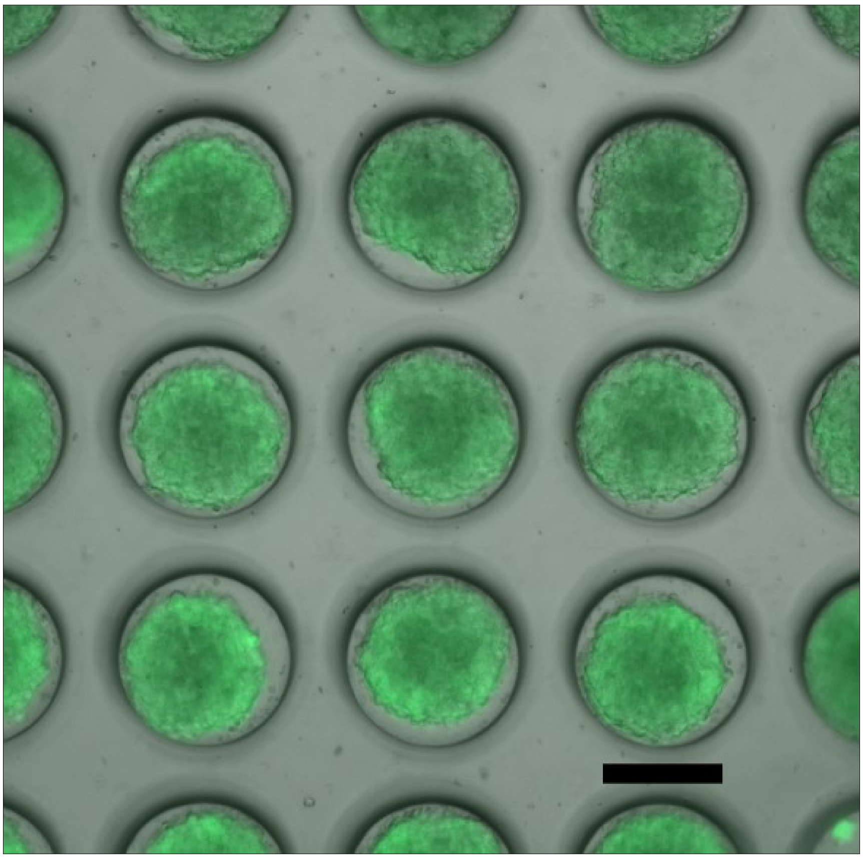

Supplementary Figure 1: Close-up of the microwell array with HEK293T cell spheroids after 3 days of culture. After depositing the cell suspension in the microwell array, cells divide over the wells and aggregate into spheroids to reach a final size that depends on the culturing time, original cell number, and cell type. Here, cells are stained with Calcein AM (AAT Bioquest) for visualization and confirmation of cell viability. The image is an overlay of a brightfield image and a fluorescence image, cropped from an original image taken with an inverted fluorescence microscope (Zeiss Axio-Observer) using a 5x/NA 0.16 air objective. Scale bar 200 µm.

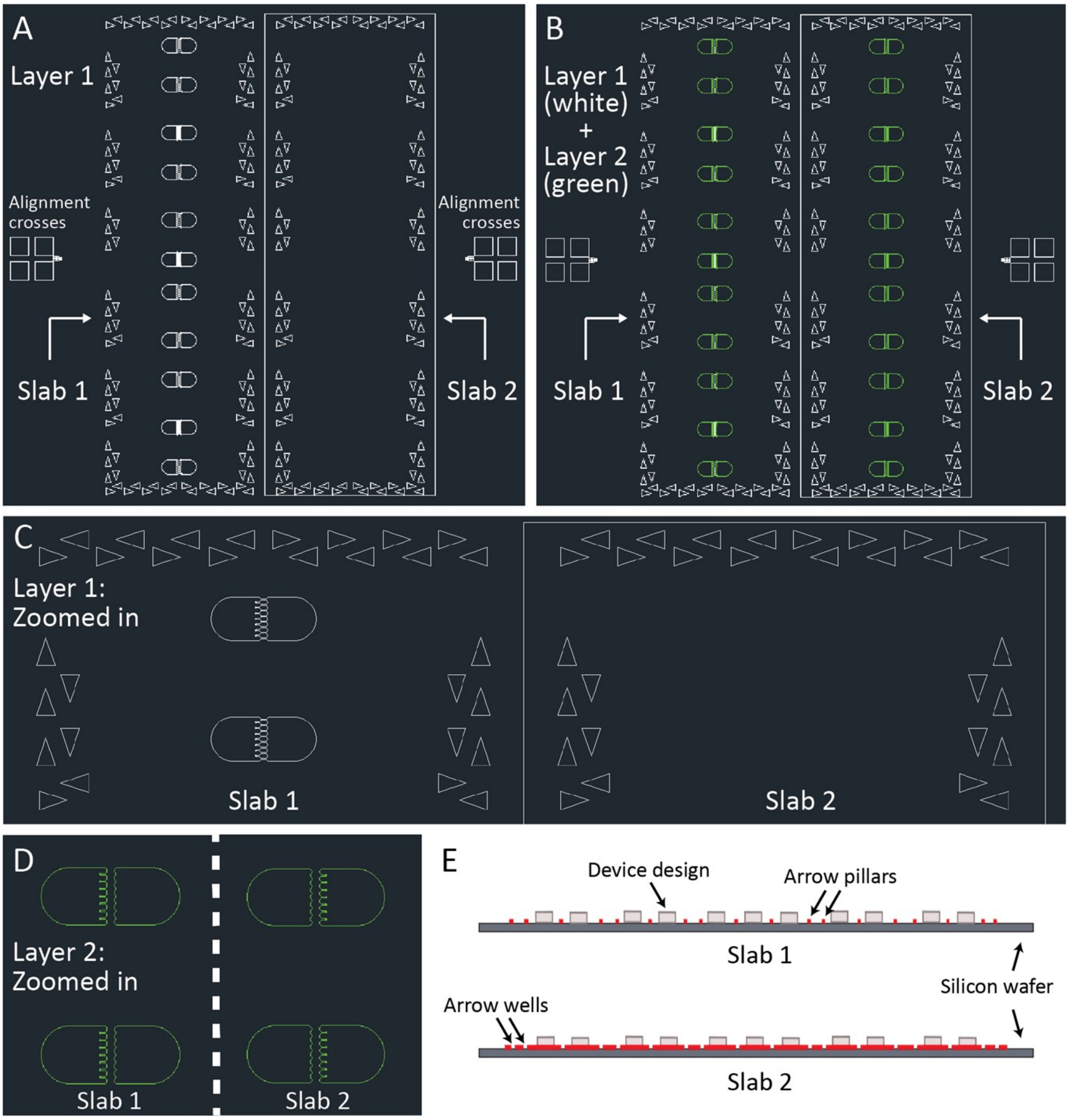

Supplementary Figure 2: Alignment of PDMS slabs using alignment arrows in the design. (A-D) AutoCAD screenshots of the full design that was printed on the silicon wafer using soft lithography. (A) First layer, which was printed to a height of ±50 µm, with the design enframed with alignment arrows. For slab 1, the alignment arrows are written by the laserwriter (µMLA, Heidelberg Instruments) as pillars, while for slab 2 an entire rectangular surface is written *except* for empty wells complementary to the arrows of slab 1. Alignment crosses are written to facilitate the alignment of layer 2 with layer 1 in the next step of the writing process. (B) Final developed design, where the second written layer is indicated in green. (C) Zoomed-in section of layer 1, clarifying the placement of alignment arrows. (D) Zoomed-in section of layer 2 (with the middle part cut out) to demonstrate how the right part of the wafer design is *mirrored* to the left part. This is necessary to create the correct final design, as PDMS slab 2 is turned over when aligning it with PDMS slab 1. (E) Side view of the final developed design on the silicon wafer, which is used for PDMS casting. Slab 1 contains arrow pillars and slab 2 complementary arrow wells (both indicated in red) to facilitate easy alignment before bonding the two slabs in order to create the final device.

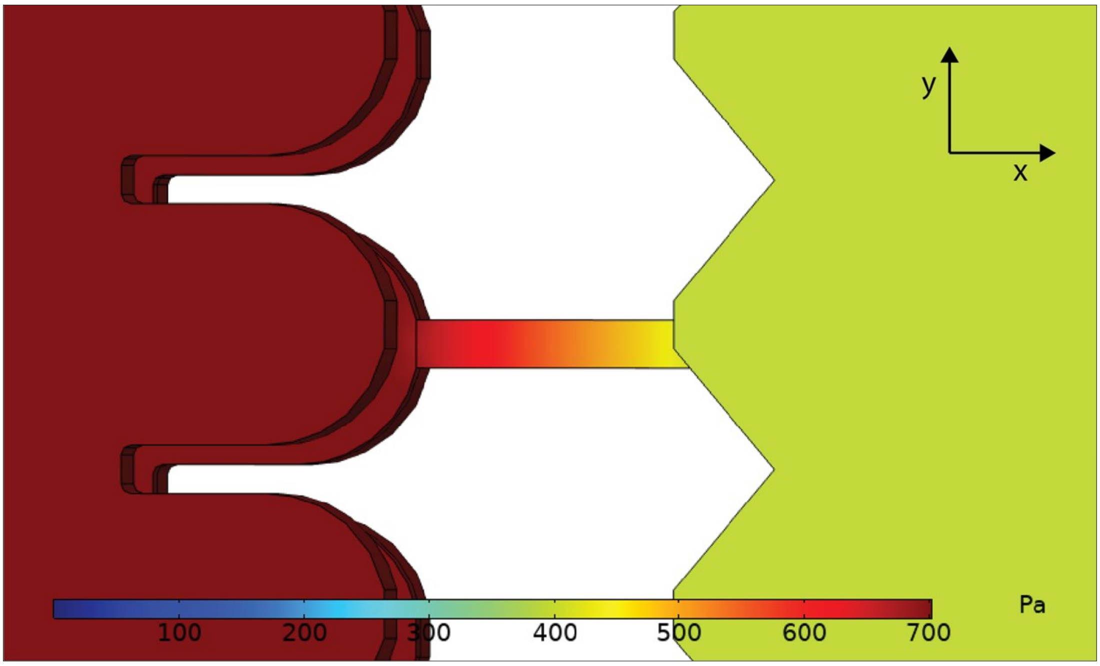

Supplementary Figure 3: Pressure distribution across aspiration channel for a microfluidic device that is misaligned 20 µm along both the x- and y-axis. A 3D numerical COMSOL simulation of the pressure distribution assuming the device is connected to a 60 cm long straight rectangular channel mimicking the outlet tube. A pressure gradient of 700 Pa is simulated for the condition that all pockets but one are clogged by spheroids. The misalignment has negligible effects on the pressure drop across the aspiration channel in comparison to a perfectly aligned chip (Fig. 3A).

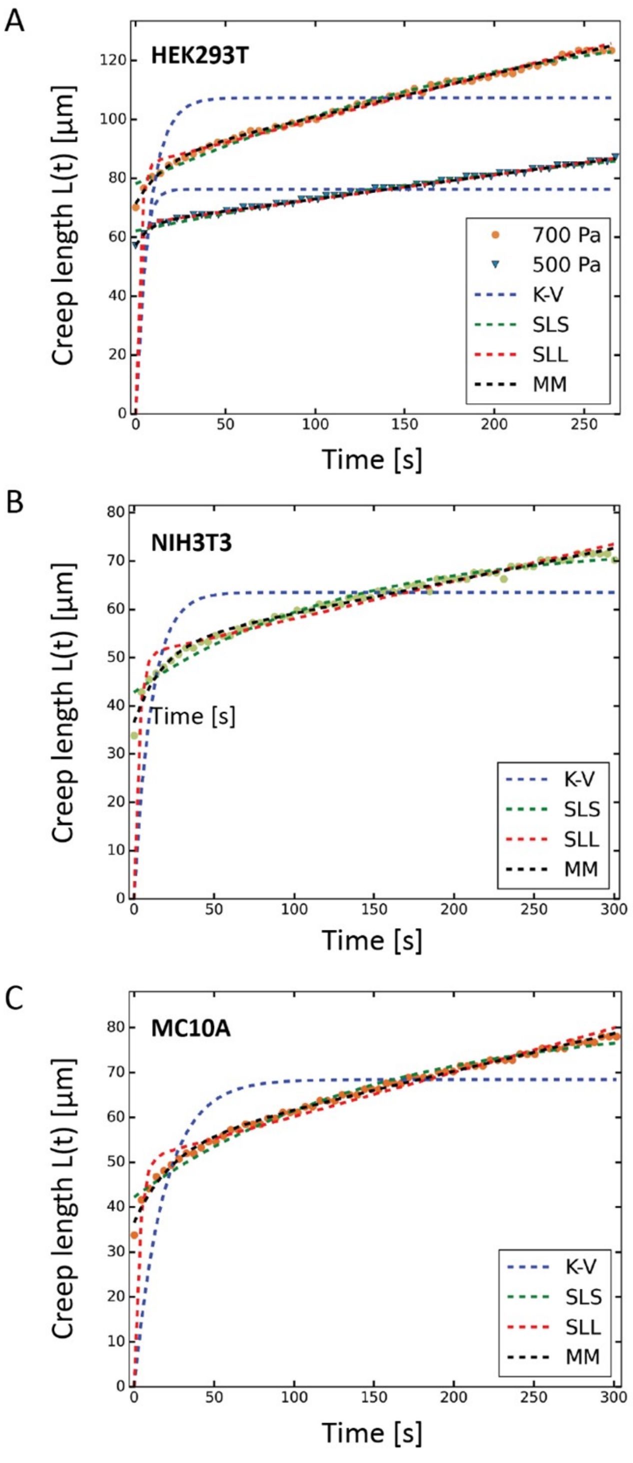

Supplementary Figure 4: Comparison between fits of different spring-and-dashpot models to the viscoelastic creep response of cell spheroids. Measured viscoelastic creep responses over time (solid circles) fitted to four different spring-and-dashpot models (represented by dashed lines), explained in the Supplementary Information, for (A) an HEK293T spheroid aspirated at 500 Pa and one at 700 Pa, (B) an NIH3T3 spheroid aspirated at 1500 Pa, and (C) an MCF10A spheroid aspirated at 1500 Pa. The Kelvin-Voigt (K-V) and Standard Linear Solid (SLS) models do not result in a viscous linear increase with time and are thus not suitable to fit the viscoelastic creep behaviour of spheroids. The Standard Linear Liquid model (SLL) is not able to account for the initial jump in L(t), unlike the modified Maxwell model (MM) due to its additional spring in the Kelvin-Voigt element. Accordingly, the R^2-values are the largest for the MM-model (see Table S1.)

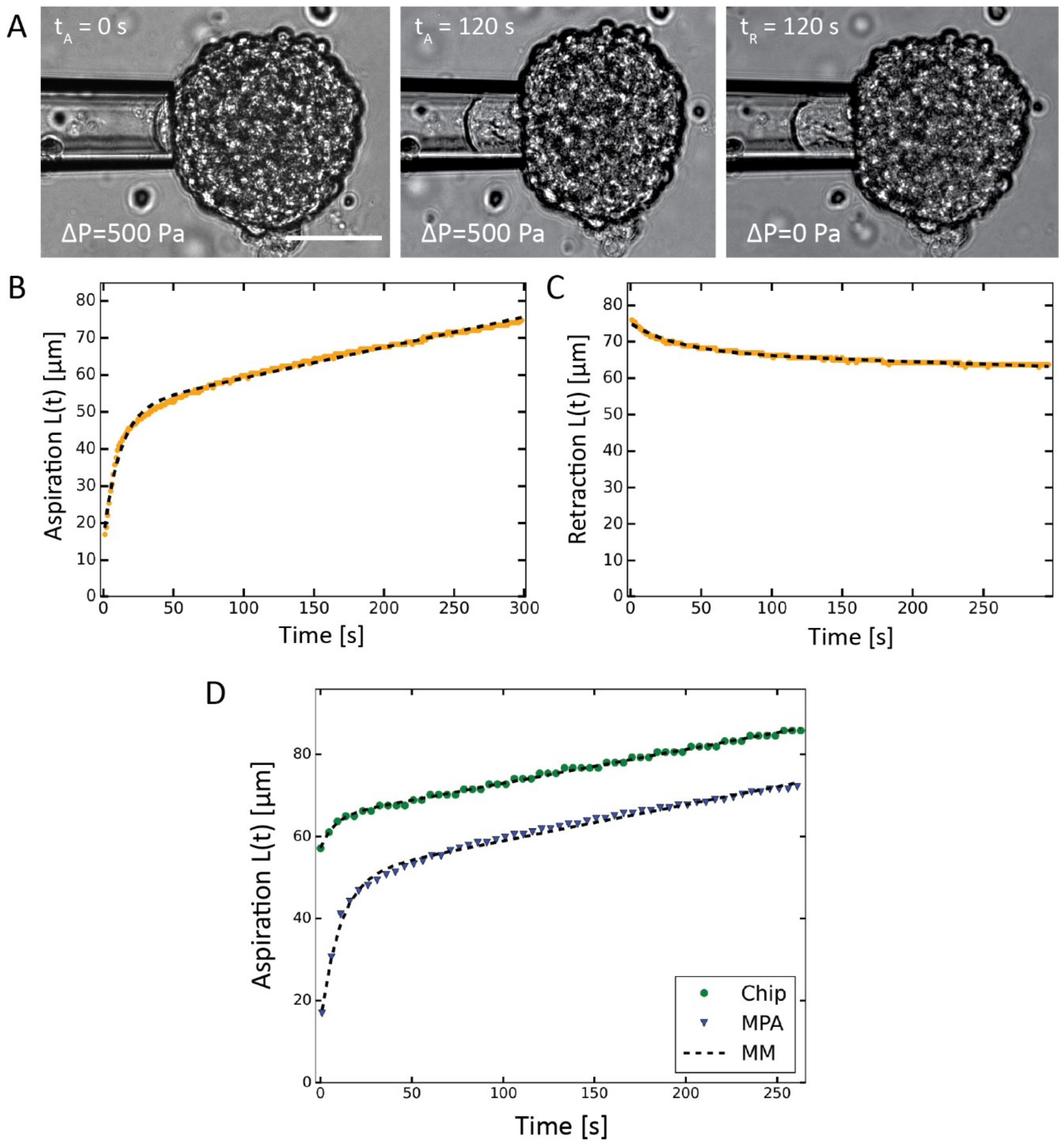

Supplementary Figure 5: Glass micropipette aspiration experiment. (A) Brightfield images of the micropipette aspiration of an HEK293T cell spheroid using a glass micropipette with a radius R_p = 32.5 µm. Left and middle panels: images taken during aspiration at a hydrostatic underpressure of ΔP = 500 Pa, at the beginning of the experiment at t_A = 0 s (left) and after t_A = 2 minutes (middle). Right image: After 5 minutes, the pressure gradient is removed and the retraction is measured, here shown after t_R = 2 minutes (right). Scale bar 100 µm. (B) Creep curve (yellow) of the aspiration of the tongue over time, and (C) creep curve (yellow) for the retraction of the tongue, both fitted with the Modified Maxwell model (black dashed lines). (D) Comparison plot of the aspiration creep curves of two HEK293T cell spheroids aspirated at ΔP = 500 Pa, one using our microfluidic device (circles) and one using the traditional glass micropipette (triangles), together with fits to the MM model (dashed lines).

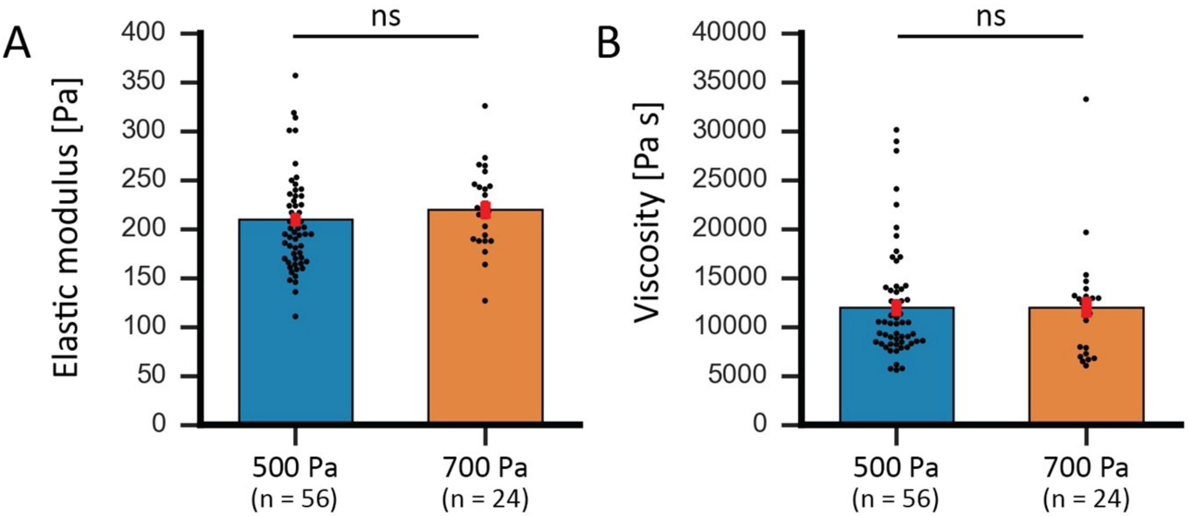

Supplementary Figure 6: Pressure independence of elastic modulus *E* and viscosity *η*. (A) Histograms comparing (A) the average elastic modulus *E* and (B) the average viscosity *η* for HEK293T cell spheroids aspirated with the microfluidic chip at different pressures (500 Pa and 700 Pa), demonstrating how the two populations are not statistically different from each other when aspirated at different pressures. ns is nonsignificant with n = 56 and 24 for 500 Pa and 700 Pa respectively. Error bars are SEM.

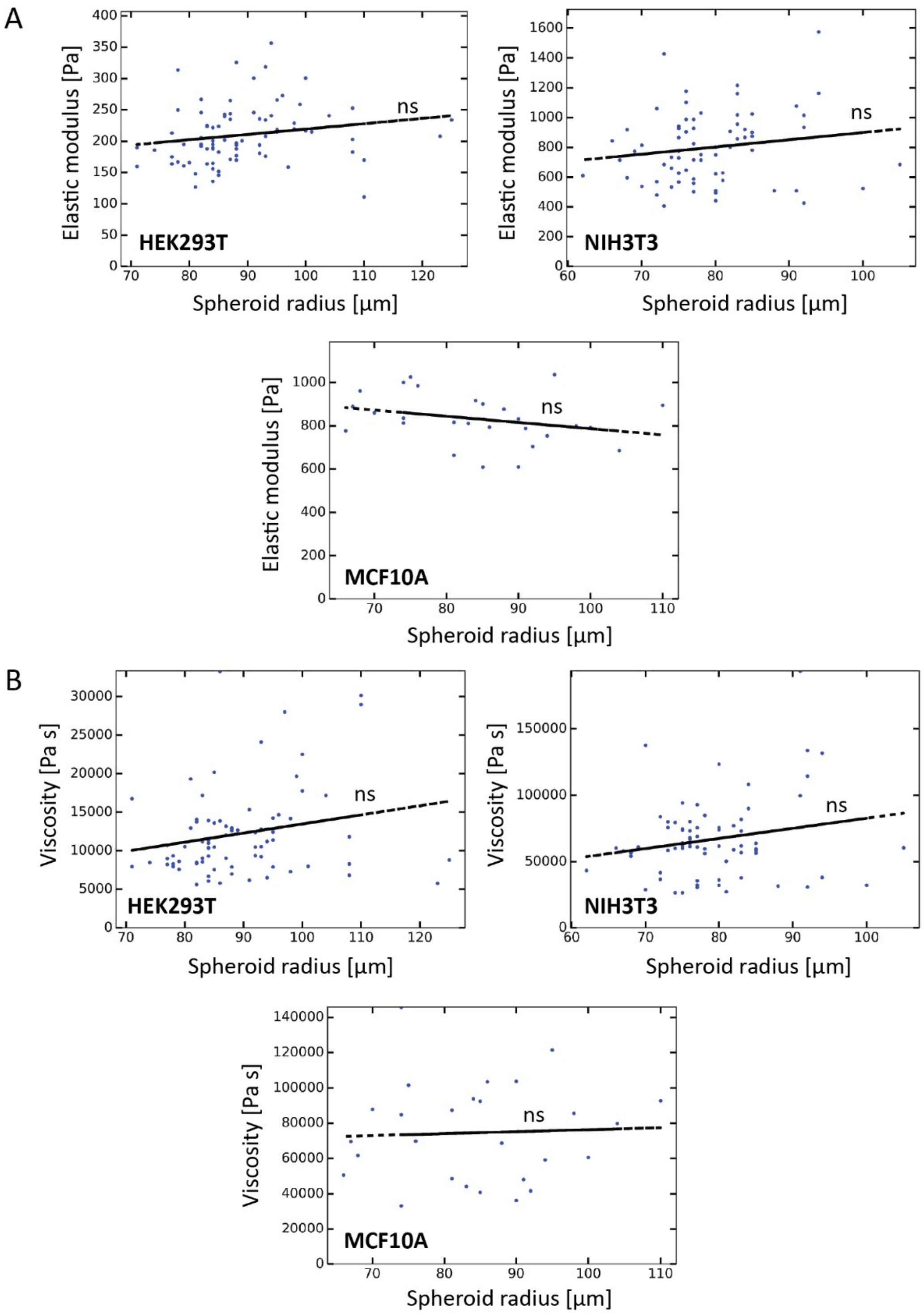

Supplementary Figure 7: Spheroid size independence of elastic modulus *E* and viscosity *η*. Scatter plots of (A) the elastic moduli and (B) viscosities for HEK293T cell spheroids (n = 80, top-left), NIH3T3 cell spheroids (n = 75, top-right) and MCF10A cell spheroids (n = 34, bottom-middle), plotted against the spheroid radius R_0 (measured with ImageJ). All plots are fitted to determine a potential linear relationship (black dashed line), ns is nonsignificant.

Supplementary Tables

Model	***Coefficient of determination (R^2)***			
	HEK293T (500 Pa)	***HEK293T (700 Pa)***	***NIH3T3 (1500 Pa)***	***MCF10A (1500 Pa)***
Kelvin-Voigt (K-V)	0.18816	0.31271	0.47405	0.58170
Standard Linear Solid (SLS)	0.98363	0.98549	0.96446	0.97898
Standard Linear Liquid (SLL)	0.65010	0.77660	0.84653	0.88069
Modified Maxwell (MM)	0.99640	0.99692	0.98872	0.99505

Supplementary Table 1: Values of the coefficient of determination (R^2) calculated for each model fitted to the viscoelastic creep response of an HEK293T cell spheroid aspirated at 500 Pa and one at 700 Pa, and an NIH3T3 cell spheroid and MCF10A cell spheroid aspirated at 1500 Pa.

Nr.	***R_0 (μm)***	***$E_{\Delta P-\Delta Pc}$ (Pa)***	***$\eta_{\Delta P-\Delta Pc}$ (kPa s)***	***$E_{\Delta P}$ (Pa)***	***$\eta_{\Delta P}$ (kPa s)***	***ΔP_c (Pa)***
1	92	317	30	383	36	86
2	76	237	21	345	31	156
3	99	328	22	419	28	109
4	101	276	18	319	21	68
5	84	281	23	364	30	113
6	87	270	26	374	37	138

Supplementary Table 2: Mechanical characteristics of HEK293T spheroids with different radii R_0 measured by glass micropipette aspiration. The elastic modulus E and viscosity η were derived from creep curves fitted to the Modified Maxwell (MM) model, either using just the applied aspiration pressure ΔP or taking the aspiration pressure corrected for the critical pressure ΔP_c (from Eq. S1 in the Supplementary Information), $\Delta P - \Delta P_c$.

Supplementary Movies

Movie 1: Microfluidic multi-channel aspiration experiment to determine spheroid mechanics.

A brightfield video of HEK293T spheroids being aspirated into the aspiration channels under an applied hydrostatic pressure of ΔP = 700 Pa for a total duration of 5 minutes. Scale bar is 200 µm.

Movie 2: NIH3T3 cell spheroids move out of pockets during retraction measurement.

A brightfield video of NIH3T3 spheroids being aspirated under a hydrostatic pressure of ΔP = 1500 Pa for the first 10 minutes, after which the pressure gradient is removed and spheroid tongues start retracting. The tongues retract within seconds, making it impossible to record a retraction curve and extract a retraction rate $\dot{L}_R$ to derive a critical pressure ΔP_c. Scale bar is 200 µm.

Movie 3: MCF10A cell spheroid retraction with remaining small aspiration pressure of 100 Pa.

A brightfield video of an MCF10A spheroid being aspirated under a hydrostatic pressure of ΔP = 1500 Pa for the first 10 minutes, after which the pressure gradient is reduced to a small remaining aspiration pressure of 100 Pa and the spheroid tongue starts retracting. The tongue again retracts rapidly, within seconds, thereby ruling out the probability that this fast retraction is caused by a backflow. Scale bar is 50 µm.

Movie 4: MCF10A cell spheroid retraction in chip without pluronic coating.

A brightfield video of an MCF10A spheroid in a chip that was *not* coated with pluronic F-127 solution, being aspirated under a hydrostatic pressure of ΔP = 1500 Pa for the first 10 minutes after which the pressure gradient is removed and the spheroid tongue starts retracting. The tongue again rapidly retracts, within seconds, thereby demonstrating that this fast retraction is probably not governed by the surface treatment of the aspiration channels. Scale bar is 50 µm.
